# Reactions of Soy Flour and Soy Protein by Non-Volatile Aldehydes Generation by Specific Oxidation

**DOI:** 10.3390/polym11091478

**Published:** 2019-09-10

**Authors:** Charles R. Frihart, Antonio Pizzi, Xuedong Xi, Linda F. Lorenz

**Affiliations:** 1 Forest Products Laboratory, USDA, 1 Gifford Pinchot Ave., Madison, WI 53726, USA; 2 LERMAB-ENSTIB, University of Lorraine, 27 rue Philippe Seguin, 88000 Epinal, France; 3 Department of Physics, King Abdulaziz University, Al Ehtifalat St, Jeddah 21589, Saudi Arabia

**Keywords:** soy flour, soy protein isolate, insoluble carbohydrates, periodate oxidation, aldehydes generation, condensation reactions, soy adhesives

## Abstract

Soy protein isolate (SPI) and insoluble soy flour polymeric carbohydrates have been reacted with sodium periodate for the specific oxidation of vicinal –OH groups to investigate the reactions involved in this approach to soy flour adhesives. The reactions have been shown to generate carbohydrate oligomer fractions presenting one, two or multiple aldehyde groups. With the exception of the small molecular weight heptanedial, the smaller molecular weight aldehydes generated from mono- and disaccharides by the same reaction do not appear to form from the insoluble soy flour carbohydrates, or have already reacted. The reaction of periodate with soy protein isolate has been shown to generate some aldehydes too. When the mix of SPI and soy insoluble carbohydrates is treated with periodate, the majority of the observed aldehyde carrying species appear to be higher molecular weight carbohydrate oligomer fractions.

## 1. Introduction

Recently, to meet new environmental standards, a number of different approaches to the use of natural materials have been used to prepare wood adhesives without the use of formaldehyde. The use of carbohydrate oxidants is a long well-known practice for a number of different applications. Thus, a number of different oxidation systems have been used to generate aldehydes from polymeric carbohydrate, the literature on this being rather abundant. These include sulfoxide-carbodiimide and related methods [1], nitroxyl radical mediated aqueous oxidation [2], special salts oxidation [3] and more recently nitro-oxidation methods [4,5], this latter being well suited for nanocellulose generation. Among these, the periodate specific oxidation of oligomeric carbohydrates is a well-known system. Thus, such a system is reported from the older periodate oxidation literature on aldehyde generation [6,7,8] to the more recent literature on nanocellulose generation by such a system followed by ozonization [9]. Among the more recent applications, the use of specific oxidants of monomeric or oligomeric carbohydrates in soy flour have shown particularly interesting results, allowing not only the protein fraction but also the carbohydrate fraction to participate positively in the preparation of wood panel adhesives [10,11,12]. In this approach, soy flour adhesives have been successfully prepared by treating soy flour with either potassium permanganate or sodium periodate [12]. Sodium periodate is a specific oxidant for carbohydrates reacting with adjacent vicinal hydroxyl groups to form dialdehydes [6,12] according to the reaction:

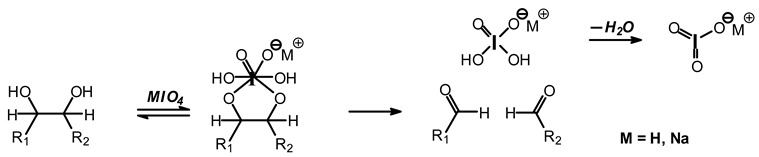



While this reaction and outcome are better known for carbohydrate monomers [6,13] and dimers [13] such as glucose and sucrose, equivalent reactions are known also for higher carbohydrate oligomers up to cellulose itself [12,14,15]. In the case of cellulose and long carbohydrate oligomers alone, the reactions that have been shown to occur are the condensation of the aldehydes formed with other carbohydrate chains to yield crosslinking, leading to solid panels in the case of cellulose [15]. By increasing the level of oxidation with further periodate in the presence of an aldehyde-reactive species such as a soy protein [12], a flavonoid tannin [13] or other reactive species, including lignin, cross-linking can also occur, leading to feasible wood adhesives [12,13].

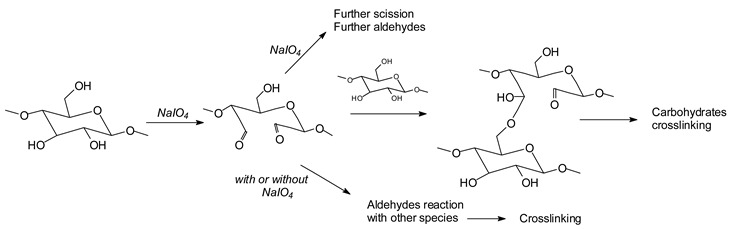



The oxidation with the periodate ion, resulting in a 1,2–glycol scission, is one of the most widely used reactions in carbohydrate chemistry. The mild reaction and the aqueous solvent conditions for periodate oxidation are particularly apt for use with water-soluble carbohydrates. The development and wide application of the reaction are due to its high degree of selectivity [6,14,15].

Aldehydes are well known for reacting with the amino group on proteins [10,11,12]. The aldehydes generated from the carbohydrate fraction of soy flour appear to react with the active sites of the soy protein, leading to cross-linking and yielding adhesives bonding plywood panels that are very encouraging for shear bond strengths, especially under wet testing conditions [12]. Equally, some of the aldehydes generated by the periodate treatment of glucose and sucrose also react with a polyflavonoid tannin, yielding equally encouraging plywood bond strengths [13].

In the case of Na periodate acting on glucose at 120 °C for 1 h, a number of different aldehydes have been shown to be generated, for example:

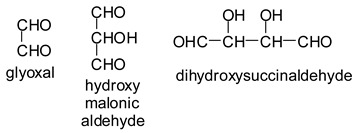



However, other aldehydes, not only from the oxidative cleavage of the carbohydrates but also via different mechanisms following the cleavage, have been shown to form [13]. The first class of these are given by the recombination of the dialdehydes obtained from the cleavage by aldol condensation [13]. Aldol condensation can occur under acid or alkaline conditions [16]. Thus, due possibly to the acid environment induced by periodate, the aldol condensation reaction starting from the glyoxal formed is for example:

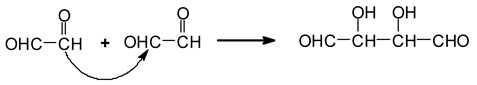



Additionally, the following species have been found to be formed via aldol condensation [13]:

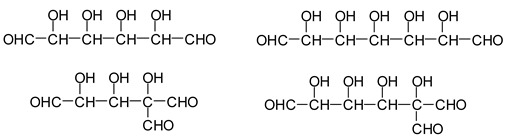



A third type of reaction has also been shown to occur due to water elimination between two aldehyde groups [13]. Aldehydes in water are in general present as hemiacetals, these being due to the reaction of an aldehyde with an alcohol or water [16]. This is the case, for example, of formaldehyde forming hemiformals in water in the preparation of formaldehyde-based resins [17].

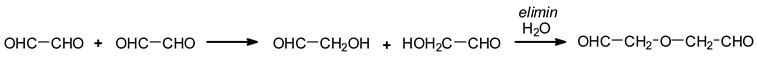



From this reaction of elimination, species such as follows were formed [3].

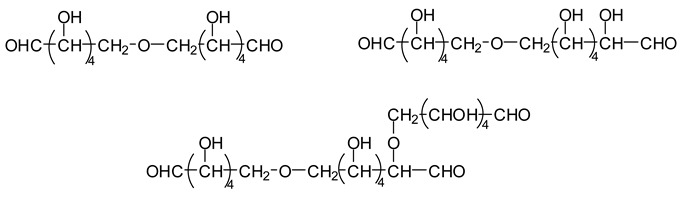



Compounds obtained by the combination of the three reactions outlined above have been shown to occur even at higher molecular weights, but aldehyde groups are always present, thus maintaining the reaction capacity of these compounds with any available reactive sites of other present compounds [13]. The reactivity of the higher molecular weight aldehydes is likely to be relatively lower than that of more active, lower molecular weight aldehydes. It must also be noted that all the aldehydes involved in subsequent reactions also have a very low volatility, eliminating the possibility of VOC emission.

However, in addition to the background given above and the reactions indicated, it has also been deduced from applied bonding results [3] that: (i) the reactions do appear to occur with soy flour, thus on insoluble polymeric carbohydrates and soluble sucrose and its alpha-galactosyl derivatives reacting, after selective oxidation, with its proteic part; and (ii) even in the absence of carbohydrates in soy protein isolates, the protein itself is able, alone, to cross-link once treated with sodium periodate, yielding encouraging plywood results. The research work presented here is then aimed to investigate (i) what aldehydes are formed via the action of periodate on the soy flour insoluble carbohydrates to explain the already experienced cross-linking [12] of periodate-treated soy flour; (ii) what occurs in the reactions of the cleaved polymeric carbohydrates with the soy protein in soy flour; and even more importantly, (iii) what happens when the soy protein isolate is treated with periodate, in the absence of carbohydrates, to make it cross-link.

## 2. Materials and Methods

### 2.1. Preparation of Samples for Analysis

In the purification of the soy protein isolate the first step involves dispersing the native flour in water at 10 percent solids, adjusting the pH to 8, centrifuging out the insoluble carbohydrates and then adjusting the pH of the insoluble carbohydrates to 6.5 [12]. (a) 10 g of isolated insoluble soy flour carbohydrates, (b) 10 g of soy protein isolate alone, and (c) 10 g of samples of a NaIO_4_-treated mix of soy protein isolate + insoluble soy flour carbohydrates + sucrose in relative proportions by a weight of 50:25:25 were all three treated with 1.5 g NaIO_4_. Controls of 10 g of soy protein isolate + 5% glutaraldehyde and 1% glutaraldehyde by weight were also prepared under the same conditions. The mixtures were placed in an oven at 120 °C for 1 h, then cooled. 

### 2.2. Matrix Assisted Laser Desorption Ionization (MALDI-TOF) Mass Spectrometry

All samples for the matrix assisted laser desorption ionization time-of-flight (MALDI-TOF) analysis were prepared by first dissolving the (a) 5 mg samples of NaIO_4_-treated insoluble soy flour carbohydrates, (b) 5 mg samples of NaIO_4_-treated soy protein flour, or (c) 5 mg samples of NaIO_4_-treated soy protein isolate alone, in 1 mL of a 50:50 *v/v* acetone/water solution. Then, 10 mg of this solution was added to 10 µL of a 2,5–dihydroxy benzoic acid (DHB) matrix. The locations dedicated to the samples on the analysis plaque were first covered with 2 µL of a NaCl solution 0.1 M in 2:1 *v/v* methanol/water, and pre-dried. Then, 1 µL of the sample solution was placed on its dedicated location, and the plaque was dried again. MALDI-TOF spectra were obtained using an Axima-Performance mass spectrometer from Shimadzu Biotech (Kratos Analytical Shimadzu Europe Ltd., Manchester, UK) using a linear polarity-positive tuning mode. The measurements were carried out making 1000 profiles per sample with 2 shots accumulated per profile. The spectrum precision is of ±1 Da.

## 3. Results and Discussion

In the case of the periodate treatment of the insoluble polymeric carbohydrates of soy flour, the cleavage of C–C bonds with vicinal –OH groups does yield aldehydes, but the peaks of the smaller aldehydes, such as glyoxal, hydroxymalonic dialdehyde and dihydroxy succinaldehyde as obtained for glucose [13] and in some cases for sucrose, are not observed, either because they are not formed (most likely due to the polymeric nature of the insoluble carbohydrates), because they are lost due to their volatile nature, or because they have already reacted in some manner. One peak already observed for glucose, given by the recombination of small dialdehydes species by aldol condensation, is nonetheless present. This sole peak, also present in glucose at 231 Da (calc. 231Da), including the Na+ enhancer, appears to belong to the action of 15% sodium periodate at 120 °C on the insoluble carbohydrates. It has already been reported for monomeric carbohydrates [13], and has the following structure:

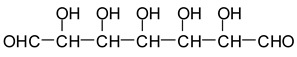



This peak, corresponding to a pentahydroxy heptanedial, is very marked when the insoluble carbohydrates are treated with periodate at 120 °C, while it is almost absent, showing just traces, when the insoluble carbohydrates alone are heated for the same period at 120 °C. It is absent at 20 °C.

**Figure 1:**
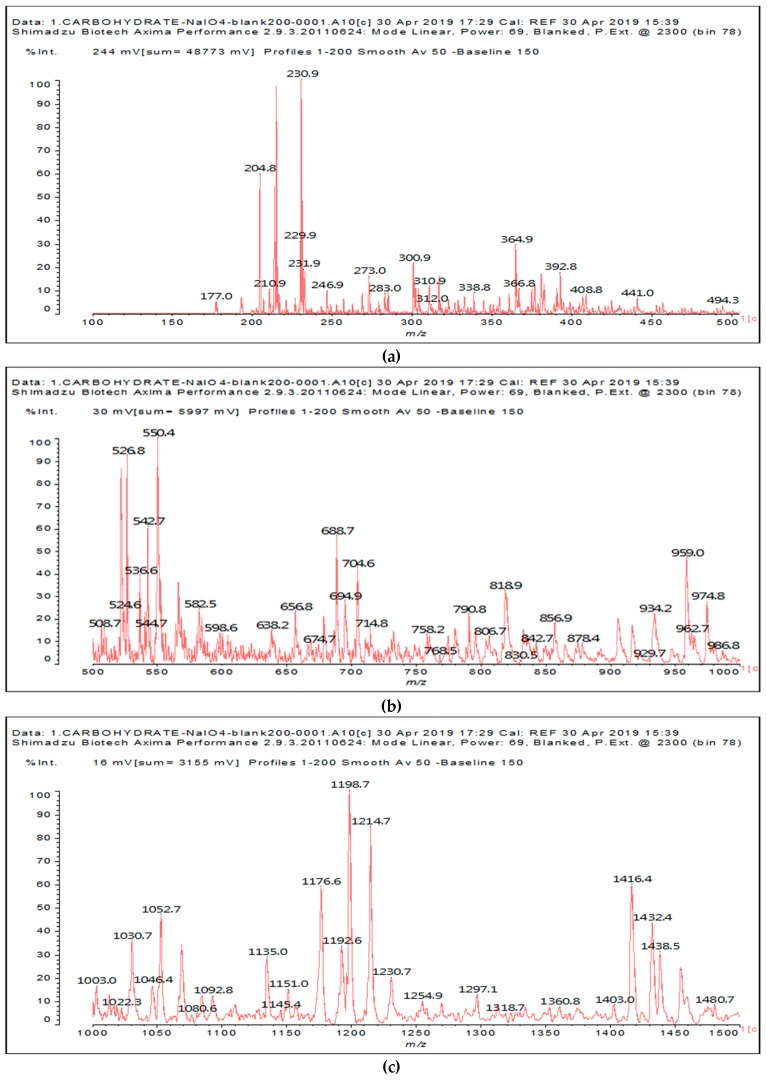
MALDI ToF spectra of insoluble soy flour carbohydrates after a treatment with sodium periodate at 120 °C for 1 h. (**a**) 100 Da to 500 Da range. (**b**) 500 Da to 1000 Da range. (**c**) 1000 Da to 1500 Da range.

The other aldehydes, found when glucose and sucrose are treated with periodate as a consequence of the other reactions outlined in the introduction, are not found for soy flour insoluble polymeric carbohydrates. Different aldehydes of much greater molecular weight are instead formed (Figure 1a–c and Table 1). Table 1 lists all the species formed, whether they carry aldehyde groups or not.

In the insoluble carbohydrates treated with sodium peroxide and in the mix proteins + carbohydrates + sucrose + periodate at 120 °C, there are a number of peaks that do not appear in the carbohydrates control at 120 °C without periodate (Supplementary Material). These are listed in Table 1, which lists all the species formed that carry aldehyde groups or not. Among these, several of the species formed after the periodate treatment are noteworthy, as they present one or more aldehyde groups. These are the peaks at 768 Da, 791 Da, 930 Da, 975 Da, 1031 Da, 1046 Da, 1053 Da, 1092 Da, 1135 Da, 1153 Da, 1176 Da, 1192 Da, 1198 Da, 1214 Da, 1230 Da, 1254 Da, 1362 Da, and 1416 Da. All present at least one aldehyde group. Among all of these there are monoaldehyde species obtained by different cleavage sites of one glucose, such as the species represented by the peaks at 768 Da, 791 Da, 1254 Da and 1416 Da:

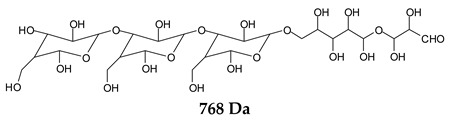

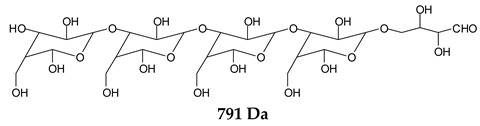





There are species presenting two aldehyde groups due to the specific oxidation of two glucoses by the periodate, such as those represented by the peaks at 1031 Da and 1053 Da:

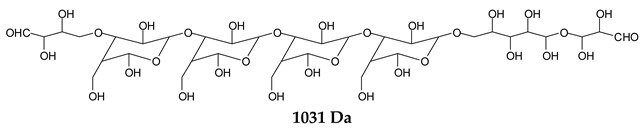



Finally, species presenting a multitude of aldehyde groups, such as several glucose pyran rings in the carbohydrate oligomer, have been cleaved by periodate. Two examples of these are the species represented by the peaks at 1214 Da and 1230 Da:

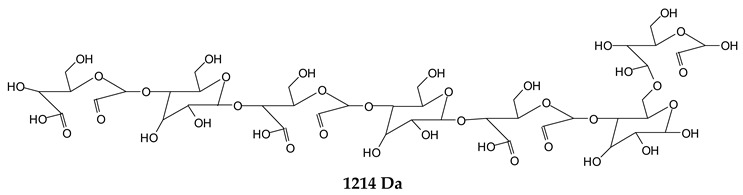

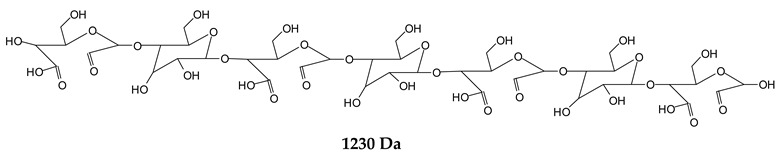





Both species confirm the reactions brought about on cellulose and other carbohydrate oligomers by previous work [6,7]. In the structures in Table 1 for the 1214 Da and 1230 Da species, the open and oxidized forms of glucose can be placed anywhere and alternate in any manner in relation to the non-oxidized glucose closed forms. Thus, the above structures and their structures in Table 1 represent just one of the possible isomers. The 1230 Da peak is separated by 16 Da from the 1214 Da peak and by 32 Da from the 1198 Da peak. This means that these two species have respectively 1 and 2 –O-atoms less than the 1230 Da one. These can have been lost in the MALDI analysis; carbohydrate chains are known to do this [18], but it could also be due to similar but slightly different species.

The marked 1198 Da peak could be a case of 6 open units linked together: 176 × 6 = 1032 + 162 units =1194 Da. It is the nearest to 1198 Da. One could also imagine that it could be a peak obtained by reactions of the small aldehydes such as glyoxal. The reactions of an aldehyde with an alcohol yields reversible hemiacetals under acid conditions. However, better and more accurate explanations are possible. Effectively, the group of peaks 1198 Da, 1214 Da, and 1230 Da are of interest due to their relative height in the high molecular weights part of the spectra in Figure 1. Of these, the 1230 Da and 1214 Da ones are interesting due to their multiple aldehyde groups. The 1198 Da species appears to be the same as the 1214 Da or the 1230 Da ones, having lost just one or two –OH groups in the MALDI analysis.

There are some peculiarities too. The peak at 1092 Da is also present in the SPI treated at 120 °C without periodate. Thus, one cannot be sure that the interpretation as an aldehyde carrying carbohydrate oligomer is correct, and a doubt must logically persist.

Monoaldehydes of higher molecular weights are also noted, such as the one at 1416 Da:

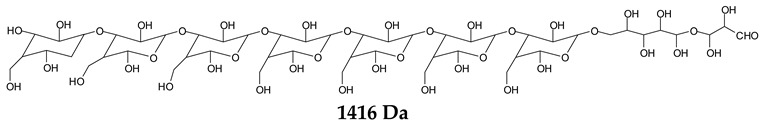



This indicates that even higher molecular weight oligomers containing aldehyde groups are likely to be generated the longer the periodate treatment is.

All of the above information indicates that the specific oxidation of the periodate appears to be able to also occur with polymeric carbohydrates, also generating aldehyde groups in high molecular weight fragments of carbohydrate chains. Consequently, they are these high molecular weight carbohydrate oligomers carrying periodate-generated aldehyde groups and the heptanedial aldehyde, which contribute to the applied bonding results with the proteic part of soy flour already reported [12]. In particular, it must be noted that species presenting a number of active aldehyde groups such as those represented by the 1230 Da and 1214 Da peaks can be a centre of tridimensional networking by a reaction with the protein, but also (as already demonstrated) by a reaction with other present carbohydrate chains [14,15].

Another interesting curiosity are the peaks at 975 Da (Calc. 976 Da) and 1135 Da (Calc. 1137 Da). The species represented by these two peaks are associated with 2 Na^+^, the 975 Da corresponding to the 930 Da peak + 2xNa^+^, and the 1135 Da peak corresponding to 1092 Da + 2xNa^+^. The 959 Da peak is the same as the 975 Da but has lost one –OH group in the MALDI analysis. The species containing two Na^+^ in the MALDI analysis are relatively unusual but are not rare and have also been reported in the previous literature [19].

**Figure 2:**
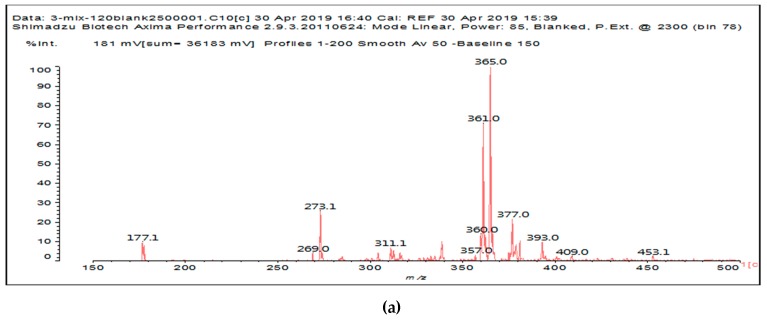

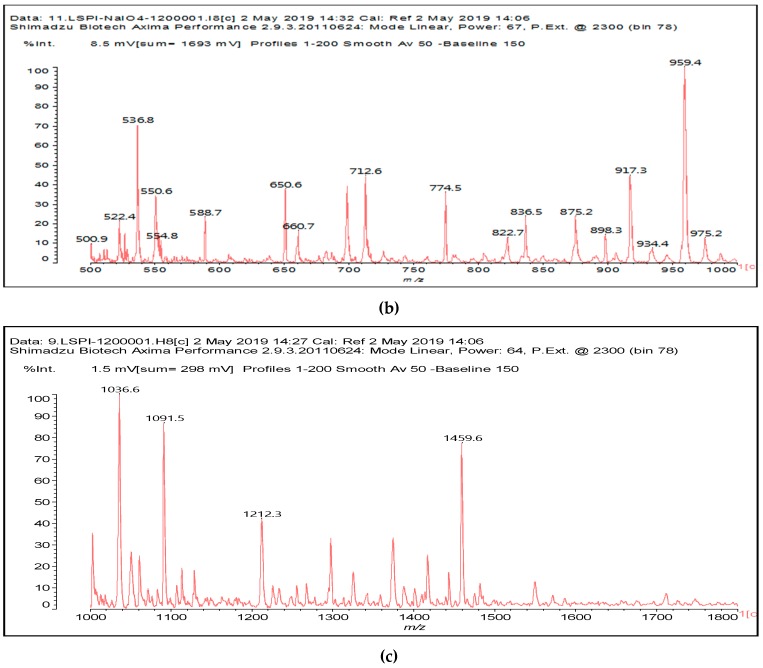
The MALDI ToF spectra of the soy protein isolate after a treatment with sodium periodate at 120 °C for 1 h. (**a**) 100 Da to 500 Da range. (**b**) 500 Da to 1000 Da range. (**c**) 1000 Da to 1500 Da range.

It is more interesting to examine the behavior of the soy protein in the soy protein isolate (SPI) when treated with sodium periodate. Notwithstanding that the amino acids and the peptides involved in these reactions are always rather difficult to identify, the task is facilitated by the fact that the relative abundance of each amino acid in the soy protein is known [20,21]. The results clearly show that the protein is also affected by a periodate treatment. Thus, on observing the peaks of the soy protein isolate + 15% peroxide present in the MALDI analysis as well as of the mix protein + soy insol. carbohydrates + sucrose + periodate, there are peaks clearly coming from the protein itself. This also shows the formation of aldehydes via the periodate oxidation of some sites of the protein itself, thus explaining why the soy protein isolate alone + peroxide also yields good bonding results without the presence of carbohydrates [12]. The main species formed that is coming from the protein are a series of monoaldehydes but also one dialdehyde, as shown in Table 1 and Figure 2a–c, the rest being difficult to assign. The question that must be answered is between which groups does the specific oxidation cleavage occur in the protein primary skeleton, as vicinal –OH carrying carbons are not present. The only possibility is that the peptidic bond itself is cleaved, as its C=O double bond is in reality delocalized with the vicinal –NH, the electronegativity being somewhat similar to that of the vicinal –OH groups.

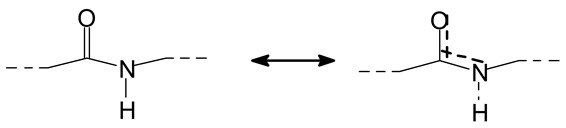



**Figure 3:**
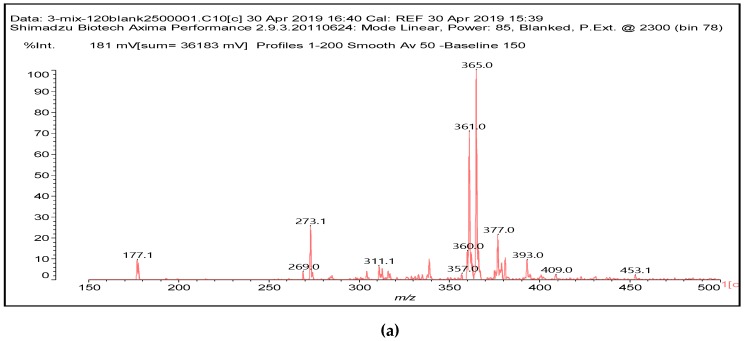

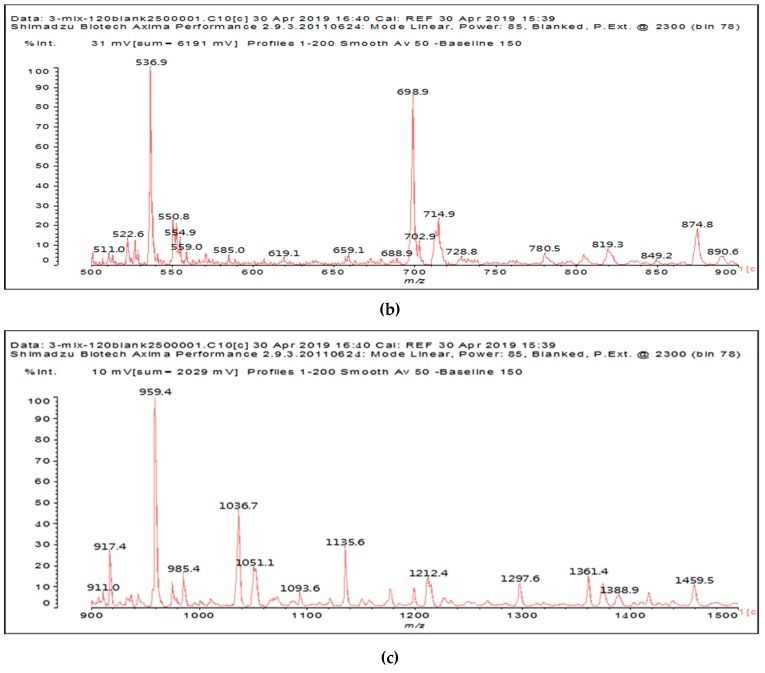
The MALDI ToF spectra of the reaction of the mix by a weight of 50% soy protein + 25% sucrose + 25% insoluble soy carbohydrates + 15% NaIO_4_ at 120 ℃ for 1 h. (**a**) 150 Da to 500 Da range. (**b**) 500 Da to 900 Da range. (**c**) 900 Da to 1500 Da range.

The location of the formed aldehyde groups seems to support such an idea. Table 2 lists the structures of the species identified according to the peaks in Figure 2 and Figure 3. Among these, several monoaldehydes are present, namely those represented by the peaks at 377 Da, 408 Da, 455 Da and the dialdehyde at 659–660 Da, namely a serine–[alanine]5–glutamic acid dialdehyde + Na+ with the following structure:

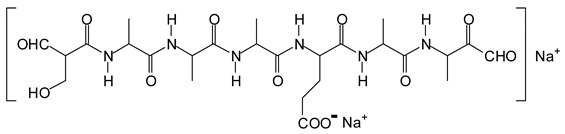



There could be several other aldehydes, but it is difficult to identify from the MALDI peaks the structures formed in the case of a protein.

It is of interest to observe the peaks present in the reaction of the reconstituted mix SPI + sucrose + soy insoluble carbohydrates + NaIO_4_ in Figure 3a–c. Among all the present peaks, the higher molecular weight (hence less reactive) aldehydes generated by sucrose oxidation are at 365 Da, 393 Da, and 523 Da. There are peaks from the protein-generated aldehydes, namely at 377 Da, 409 Da, 453 Da, and 659 Da, and peaks from protein fragments without aldehyde groups, such as at 959 Da. The generated aldehydes by oxidation of the insoluble soy carbohydrates are represented by the peaks at 689 Da, 1036 Da, 1093 Da, 1135 Da, 1212 Da, and 1361 Da and the non-aldehyde fragment at 1297 Da. The DHB matrix enhancer are represented by the peaks at 312 Da, 361 Da, 536 Da, 550 Da and 698 Da. The question is now about which peaks that are present are due to the reaction of an aldehyde, for which it does not matter how they were generated, and which are due to a reaction with amino acids. First of all, it is clear that at a high molecular weight range, many species that are formed will be out of the possible range. Second, the majority of species generated by a reaction with the amino acids of aldehyde-generated species will be found in the middle of the examined molecular weight range. Thus, the co-reacted species are those represented by the peaks that do not belong to any of the single components at 555 Da, 559 Da, 585 Da, 619 Da, 729 Da, 780 Da, 819 Da, 849 Da 890 Da, 1389 Da and 1459 Da. These are due to the reaction of some generated aldehydes with some amino acids, and their assignments are given in Table 3. The more interesting interpretable peak is the one at 585 Da. This is obtained by the reaction of the sucrose-generated tri-aldehyde at 177 Da. While this peak is also present in SPI alone representing just an amino acid, it is also a branched aldehyde generated from sucrose via the aldol condensation of simpler sucrose-generated aldehydes. Thus, the 585 Da peak is due to the reaction of three molecules of the most abundant SPI amino acid, namely glutamic acid, with:

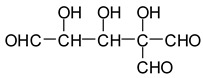



To yield the two alternative structures at 585 Da, one is derived directly by the reaction of three molecules of glutamic acid with the 177 Da tri-aldehyde and the second one is generated via the reaction of three glutamic acid residues with glyoxal and a hydroxyl malonic aldehyde directly generated by the specific oxidation cleavage by the periodate:

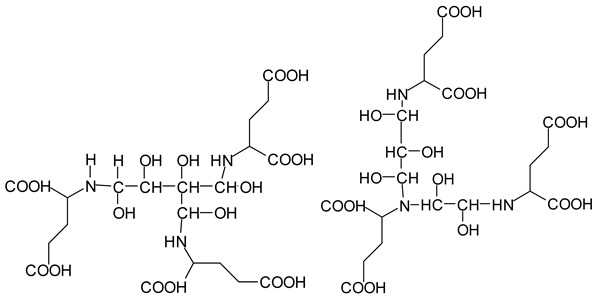



The same peak at 585 Da can also be assigned to an oligomer of:

Lysine-CHOH-CHOH-Lysine-CHOH-CHOH-CHOH-Lysine

As amino acids presenting at least two amino groups can form linear oligomers, the peaks at 554–555 Da, 619 Da, 728 Da, 780 Da, 819 Da, and 849–850 Da can be interpreted as linear oligomers of the reaction of (mainly) lysine and arginine, with glyoxal and hydroxy malonic aldehyde generated by periodate-specific oxidation from simple sugars such as glucose [3] and sucrose, as is indeed the case for one of the assignments for the species at 585 Da (Table 3).

## 4. Conclusions

To summarize, in the spectra of the reaction of SPI + sucrose + insoluble carbohydrates + NaIO_4_, the following peaks ascribed to the action of periodate on SPI and insoluble soy flour carbohydrates are present: 1053 Da, 1092–1093 Da, 1135 Da, 1153 Da, 1198 Da, 1212–1213 Da, 1230 Da, 1254 Da, 1361–1362 Da and 1416 Da coming from the insoluble carbohydrates, and 377 Da, 408–409 Da, 453–455 Da and 659–660 Da coming from the SPI. This shows that some of the carbohydrate aldehyde species are no longer present when the SPI is present; hence, they have reacted with the SPI. Furthermore, the co-reacted species are those represented by the peaks that do not belong to any of the single components, at 555 Da, 559 Da, 585 Da, 619 Da, 729 Da, 780 Da, 819 Da, 849 Da 890 Da, 1389 Da and 1459 Da.

The peak at 231 Da represents just traces (almost non-existent) in the insoluble carbohydrates at 120 °C without periodate, but it is a huge peak in the insoluble carbohydrates with periodate at 120 °C. It does not seem to appear in the SPI + sucrose + insoluble carbohydrates + NaIO4. It could be that with the protein present, the aldehyde groups of the species 231 Da have reacted very readily with SPI. It is one of the best indications that the carbohydrates have not only formed an aldehyde (double here) but also that this reacts with the SPI.

The following peaks coming from the periodate cleavage of the protein are present: 377 Da, 408–409 Da, 455 Da and 659–660 Da. Of these, the 408–409 Da, 455 Da and 659–660 Da could present an aldehyde function due to the cleavage but could also be interpreted otherwise. However, they are most likely carrying aldehyde groups, as without periodate these peaks do not seem to appear. However, the peaks at 408–409 Da and 453–455 Da also appear, although small, in the case of the carbohydrates at 120 °C without periodate, and in the SPI alone at 120 °C without periodate; thus, their interpretation in favour of aldehyde formation is not at all certain. Thus, one cannot be sure at all that they come from the protein.

The final conclusions for the reactions at 120 °C with and without periodate are then as follows:

1. There are clear indications that aldehydes are formed from the insoluble carbohydrates by periodate specific oxidation and cleavage. There are carbohydrate oligomers presenting single, double and multiple aldehyde groups coming from the cleavage of the soy flour insoluble carbohydrates.
2. There is only one, different, low molecular weight aldehyde at 231 Da, which is in considerable proportion when the insoluble carbohydrates are treated with periodate but only in traces when treated at 120 °C without periodate. This peak disappears completely when the SPI is present, indicating that the aldehyde (a dialdehyde) has reacted with the SPI.
3. The peak at 231 Da is a dialdehyde derived by the aldol condensation of fragments issued by the degradation of soluble and insoluble carbohydrates.
4. There are oligomeric peptides coming from the cleavage of the protein by periodate. However, only one species (at 659–660 Da) could (we stress could) be ascribed to an oligomeric aldehyde, as other interpretations, such as peptide oligomers without an aldehyde group, are also possible.
5. Thus, the clear indications are that the carbohydrates do form aldehydes that co-react with the SPI and crosslink it.
6. There is at least one indication that aldehyde groups are formed in the cleavage of the protein, but these are not clear and could also be interpreted otherwise.
7. In the SPI + sucrose + insoluble carbohydrates + NaIO_4_, several aldehydes are formed, as presented in the Introduction and already reported in the literature [5].
8. In the SPI + sucrose + insoluble carbohydrates + NaIO_4_, mainly aldehydes generated from sucrose by periodate-specific oxidation react with amino acids, showing that the cross-linking of the protein oligomers by these aldehydes does indeed occur. This entails that at a higher temperature or longer reaction times, the other aldehydes generated from the insoluble carbohydrates and from the protein are also likely to react and contribute to cross-linking.
9. Finally, it must be noted that the peaks at 177 Da, 360–361 Da, 536 Da, 550 Da, 698–699 Da, 717 Da, and 874 Da belong to the DHB enhancer used in the MALDI analysis.

What are the future prospects of this type of approach? The first interest is that an almost classical reaction and cross-linking system based on aldehydes and a protein are generated, but with the generated aldehydes being non-volatile and non-toxic. The second interest is that all parts of soy flour, much cheaper than soy protein isolates, can be directly used for adhesives and can participate in cross-linking. Third, while one can use soy flour, nothing impedes the use of a separate mix of oligomeric and or monomeric carbohydrates to generate aldehydes via periodate specific oxidation and then adding this to a soy protein isolate, another protein isolate (such as gluten for example), or other aldehyde-reacting materials. In this respect, periodate oxidized carbohydrates could be used as hardeners for a variety of different natural materials, such as tannins, lignin, humins, etc., or possibly even mixtures of these.

## Figures and Tables

**Table 1:** The proposed oligomer species for the MALDI ToF spectra peaks in Figure 1a–c for the reaction at 120 °C for 1 h of sodium periodate with insoluble soy flour carbohydrates. Note: the links between the glucose units are alpha-glucosidic. They are represented as being beta glucosidic to shorten the table.

231 Da (with Na^+^)
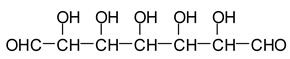
365 Da (calc. 365 Da) (with Na^+^)
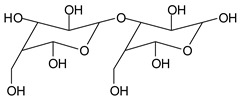
527 Da (calc. 527 Da) (with Na^+^)
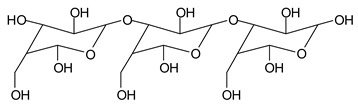
768 Da (calc.766 Da) (with Na^+^)
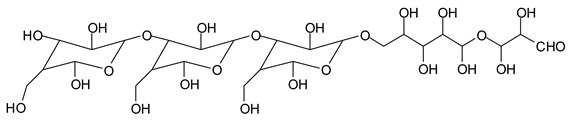
689 Da (calc. 689 Da) (with Na^+^)
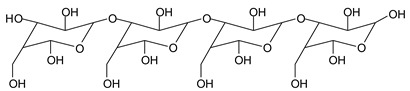
768 Da (calc. 768 Da) (no Na^+^), 791 Da (calc. 791 Da) (with Na^+^)
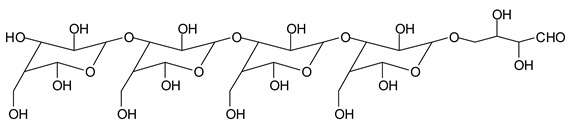
930 Da (calc. 928 Da) (with Na^+^)
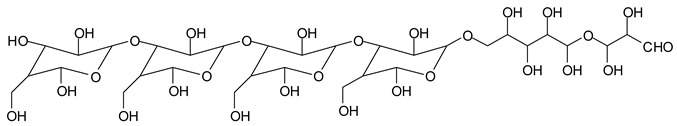
930 Da (calc. 930 Da) (no Na^+^)
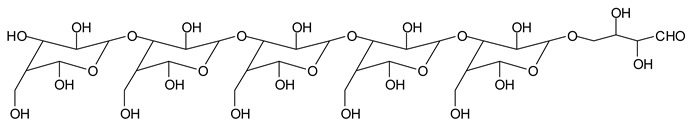
975 Da (calc. 976 Da) (= 930 Da but with 2xNa^+^) 1031 Da (calc. 1032 Da) (no Na^+^) 1053 Da (calc. 1054 Da) (with Na^+^)
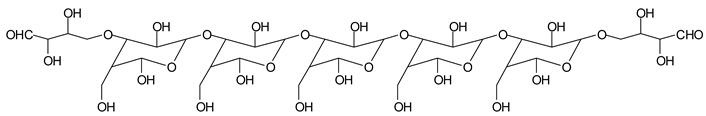
1031 Da (calc. 1029 Da) (with Na^+^)
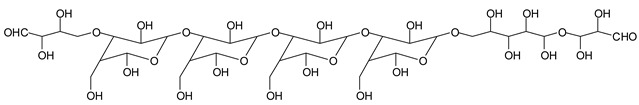
1046 Da (calc. 1043 Da) (with Na^+^)
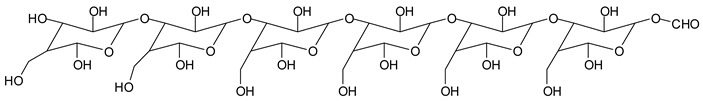
1092 Da (calc.1089 Da) (with Na^+^)
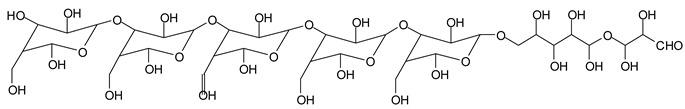
1092 Da (calc.1092 Da) (no Na^+^)
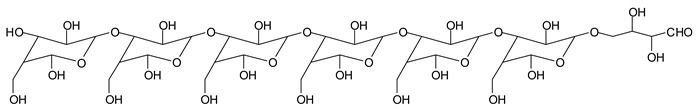
1135 Da (calc. 1136 Da) (= 1092 Da but with 2x Na^+^) 1153 Da (calc. 1153 Da) (no Na^+^) 1176 Da (calc. 1176 Da) (with Na^+^)
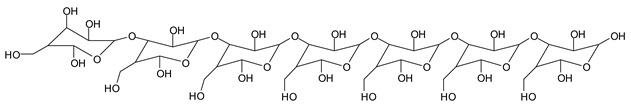
1192 Da (calc. 1192 Da) (with Na^+^)
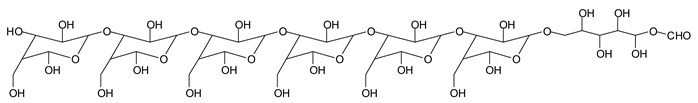
1214 Da (calc. 1212 Da) (no Na^+^)
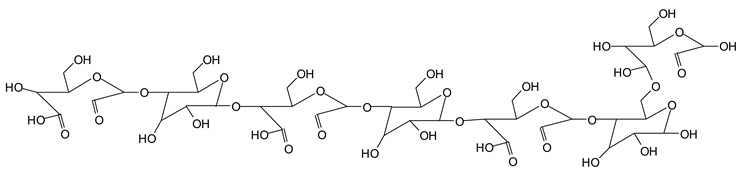
1230 Da (calc. 1231 Da) (with Na^+^)
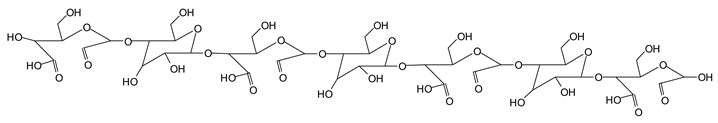
1254 Da (calc. 1253 Da) (with Na^+^)
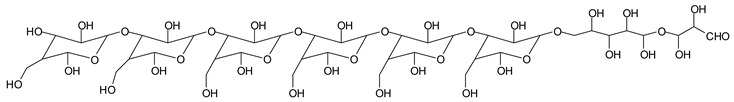
1297 Da = glucose octamer missing one –OH, (no Na^+^) 1362 Da (calc. 1364 Da) (with Na^+^)
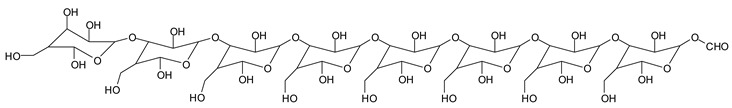
1416 Da (Calc. 1415 Da)
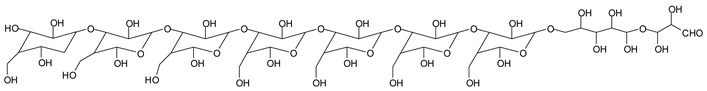

**Table 2:** The proposed oligomer species for the MALDI ToF spectra peaks in Figure 2a–c and Figure 3a,b for the reaction at 120 °C for 1 h of sodium periodate with the soy protein isolate.

377 Da = [alanine]_4_-aspartic acid **monoaldehyde** without Na^+^ 409 Da (calc. 409 Da) = alanine pentamer **monoaldehyde** with Na^+^
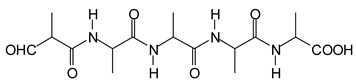
But it could also be (but less likely) 409 Da = tyrosine-triptophane + Na^+^ (dimer) 455 Da (calc. 457 Da) = alanine hexamer **monoaldehyde** without Na^+^
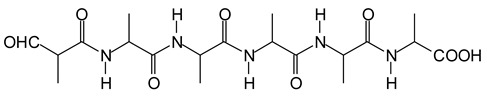
OR455 Da (calc. 457 Da) = [alanine]_2_-[serine]_3_ **monoaldehyde** + Na^+^
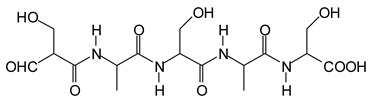
659–660 Da = phenylalanine-triptophan-leucine-histidine + Na^+^ (tetramer) 659–660 Da = phenylalanine-triptophan-histidine-aspartic acid + Na^+^ (tetramer) 659–660 Da = serine-[alanine]_5_-glutamic acid dialdehyde + Na^+^
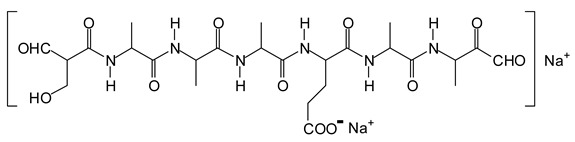
959 Da = phenylalanine-triptophan-leucine-histidine-phenylalanine-arginine + Na^+^ (hexamer) 959 Da = phenylalanine-triptophan-histidine-aspartic acid-phenylalanine-arginine + Na^+^ (hexamer)

**Table 3:** Some possible assignments from the MALDI ToF spectra in Figure 3a–c of the reaction of SPI + sucrose + insoluble soy carbohydrates + NaIO4 for peaks not belonging to any of the single periodate-treated constituent components.

554–555 Da, no Na^+^ Lysine–CHOH–CHOH–Lysine–CHOH–CHOH–Lysine
585 Da, no Na^+^, two possible species from glutamic acid
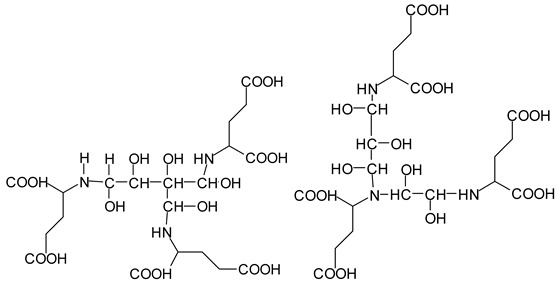
and/or one from Lysine Lysine–CHOH–CHOH–Lysine–CHOH–CHOH–CHOH–Lysine
619 Da, no Na+ Lysine–CHOH–CHOH–CHOH–Lysine–CHOH–CHOH–CHOH–Lysine
728 Da, no Na+ Lysine–CHOH–CHOH–Lysine–CHOH–CHOH–Lysine–CHOH–CHOH–Proline
780 Da, no Na+ Lysine–CHOH–CHOH–Lysine–CHOH–CHOH–Lysine–CHOH–CHOH–Phenylalanine
819 Da, no Na+ Arginine–CHOH–CHOH–Arginine–CHOH–CHOH–Arginine–CHOH–CHOH–Cysteine
849–850 Da, no Na+ Lysine–CHOH–CHOH–CHOH–Lysine–CHOH–CHOH–CHOH–Lysine–CHOH–CHOH–CHOH–Lysine and/or Lysine–CHOH–CHOH–CHOH–Lysine–CHOH–CHOH–CHOH–Lysine–CHOH–CHOH–CHOH–glutamic acid
985 Da, no Na+ Lysine–(–CHOH)_3_–Lysine–(–CHOH)_3_–Lysine–(–CHOH)_3_–Lysine CHOH–CHOH–Glycine
1388–1389 Da Arginine–(–CHOH)2–Arginine–(–CHOH)2–Arginine–(–CHOH)2–Arginine–(–CHOH)2–Arginine–(–CHOH)2–Tryptophan Where the structure is not necessarily linear and can be better expressed as [Arginine–(–CHOH)_2_–]_5_–Tryptophan
1459–1460 Da [Arginine–(–CHOH)_2_–]_5_–Histidine–(–CHOH)_4_–CHO or [Arginine–(–CHOH)_2_–]_5_–Histidine–(–CHOH)_2_–Alanine

## Supplementary Materials

The following are available in the Supplementary Material online at https://www.mdpi.com/2073-4360/11/9/1478/s1, Figure S1: MALDI Tof Spectra of insoluble carbohydrates treated at 120 °C without periodate: (a) 150 Da to 500 da range, (b) 500 Da to 900 Da range, and (c) 900 Da to 1200 Da range. Figure S2: sucrose + 15% NaIO_4_ at 120 °C for 1 h: (a) 50 Da to 1000 Da range, (b) 50 Da to 500 Da range, (c) 300 Da to 500 Da range, (d) 500 Da to 800 Da range, and (e) 800 Da to 1600 Da range.
