# Supplementary material for: Reactions of Soy Flour and Soy Protein by Non-Volatile Aldehydes Generation by Specific Oxidation
Source: Polymers (Basel). 2019 Sep 10;11(9):1478. doi: 10.3390/polym11091478 (PMC6780918; doi:10.3390/polym11091478)
Supplement: Supplementary file 1 [file polymers-11-01478-s001.pdf]

SUPPLEMENTARY MATERIAL

Figure S1: Insoluble Carbohydrates 1h at 120°C (no peroxide). (a) 150 Da to 500 Da range. (b) 500 Da to 900 Da range. (c) 900 Da to 1200 Da range.

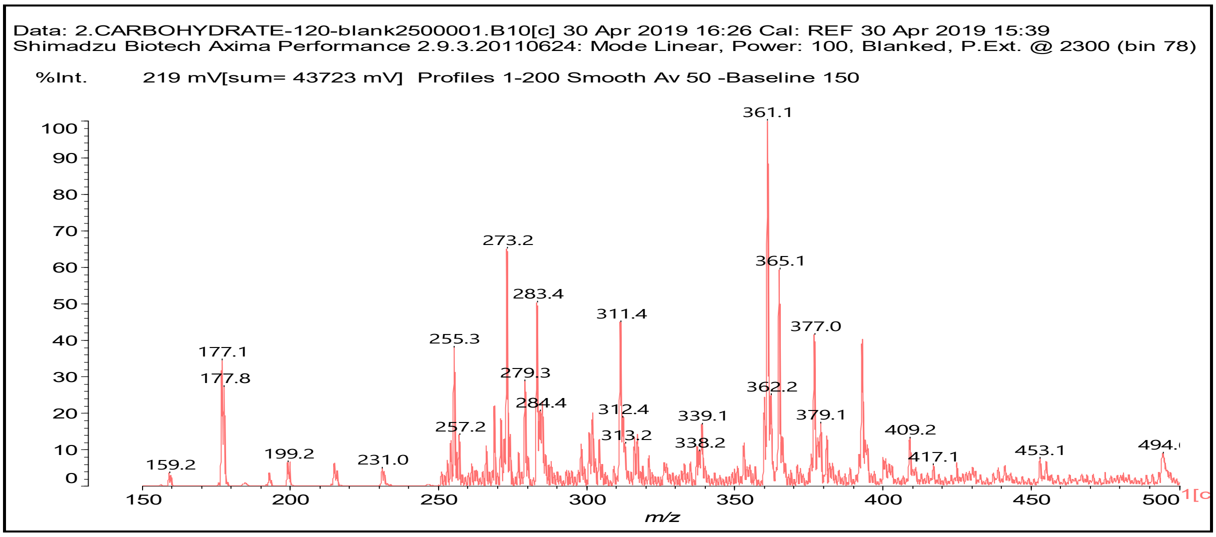

(a)

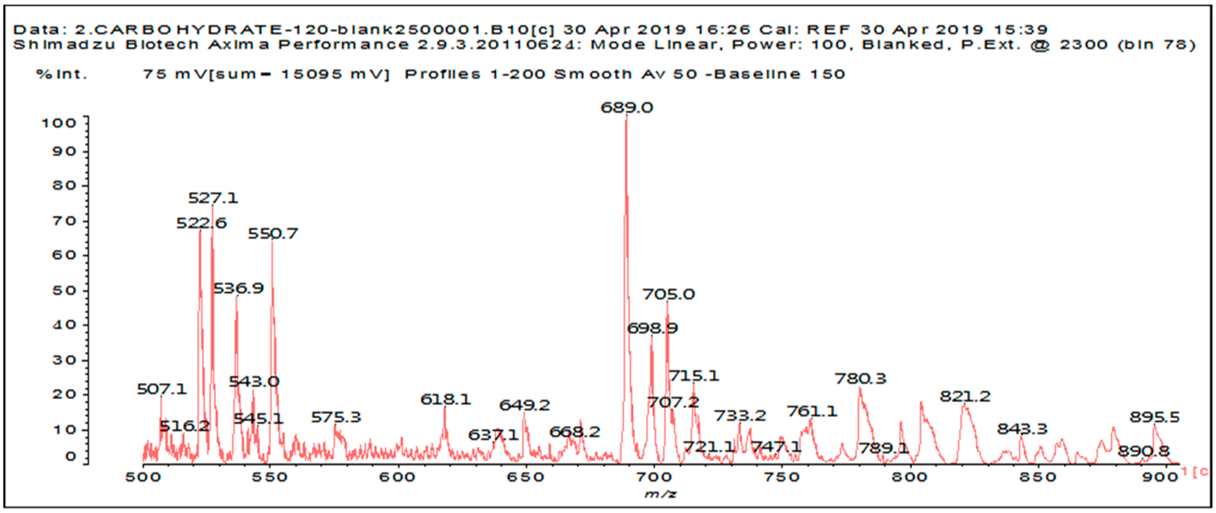

(b)

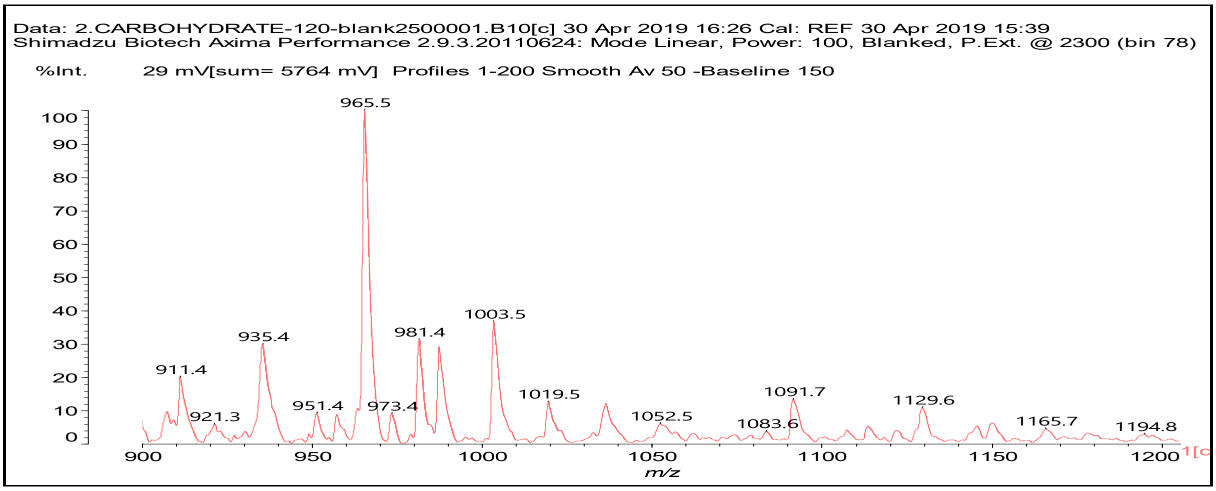

(c)

Figure S2 : sucrose+15%$NaIO_4$ at 120°C for 1 hour.). (a) 100 Da to 1000 Da range. (b) 50 Da to500 Da range. (c) 300 Da to 500 Da range. (d) 500 Da to 800 Da range. (e) 800 Da to 1600 Da range.

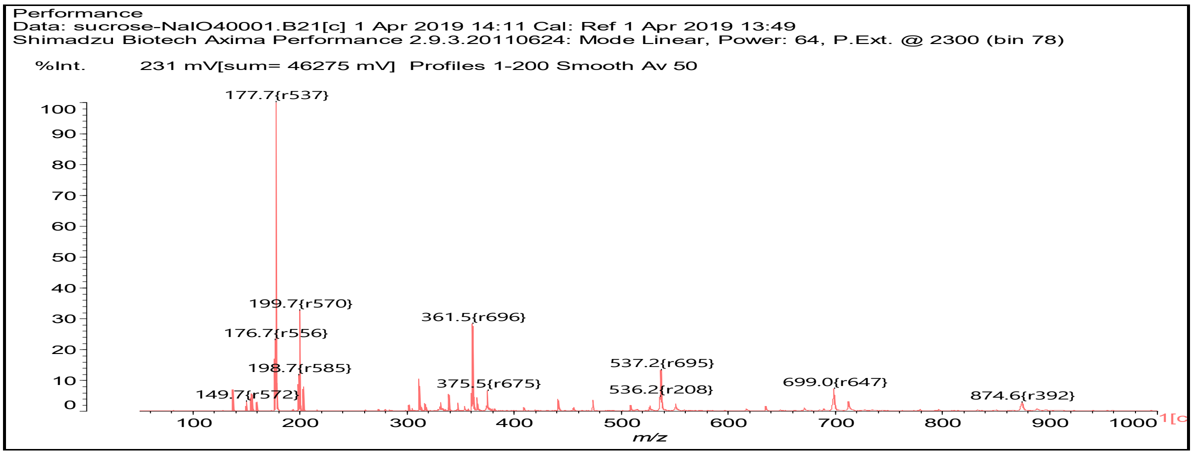

(a)

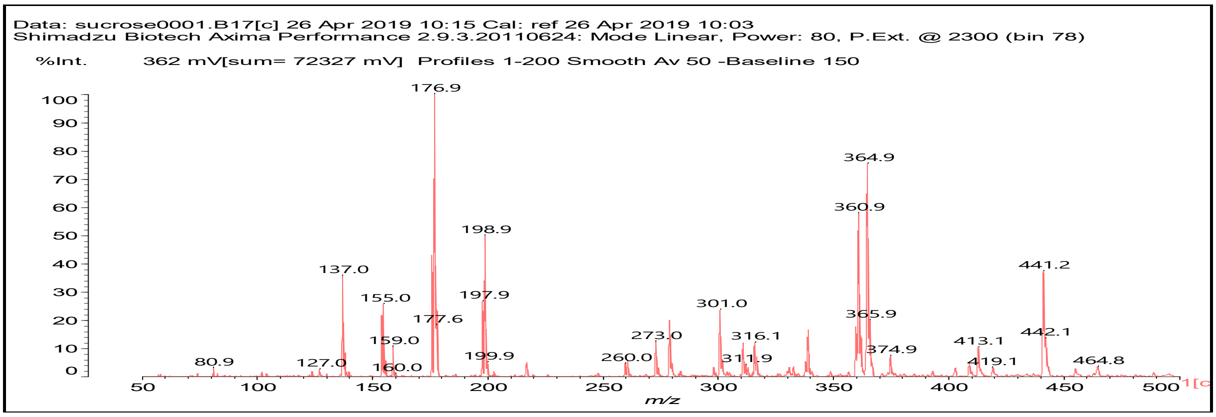

(b)

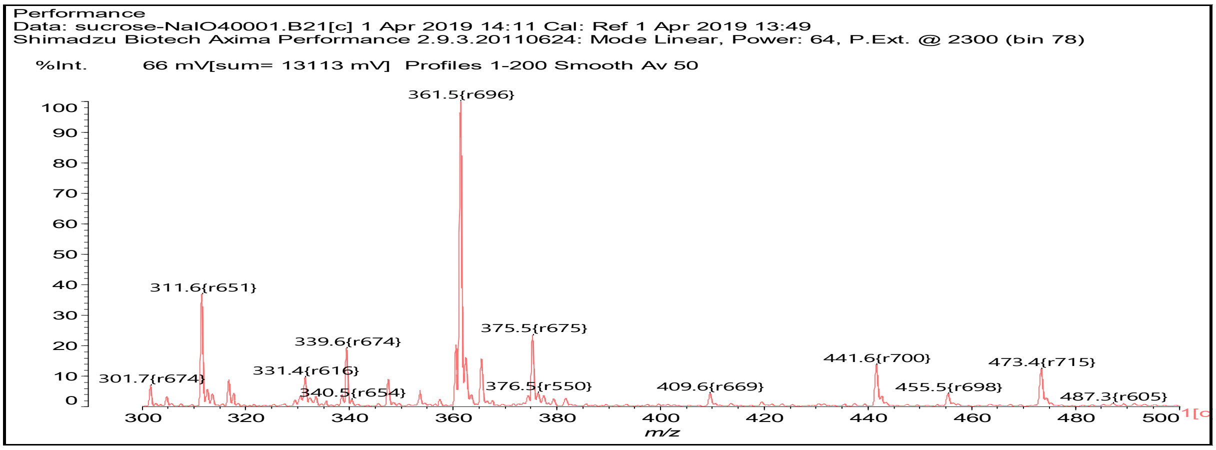

(c)

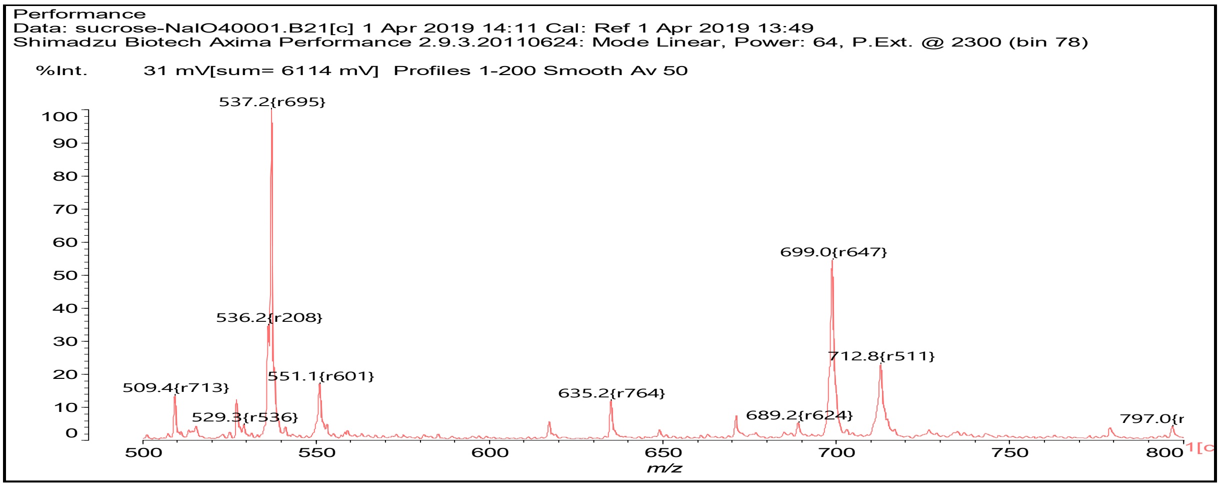
Performance
Data: sucrose-NaIO40001.B21[c] 1 Apr 2019 14:11 Cal: Ref 1 Apr 2019 13:49
Shimadzu Biotech Axima Performance 2.9.3.20110624: Mode Linear, Power: 64, P.Ext. @ 2300 (bin 78)
%Int. 31 mV[sum= 6114 mV] Profiles 1-200 Smooth Av 50
537.2{r695}
699.0{r647}
536.2{r208}
712.8{r511}
551.1{r601}
509.4{r713}
635.2{r764}
529.3{r536}
689.2{r624}
797.0{r
m/z

(d)

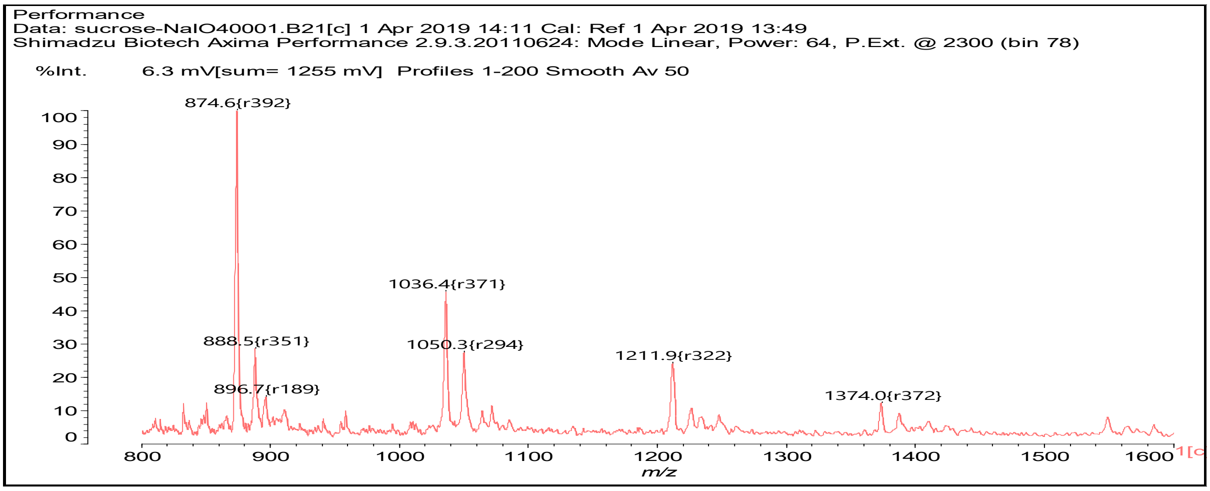
Performance
Data: sucrose-NaIO40001.B21[c] 1 Apr 2019 14:11 Cal: Ref 1 Apr 2019 13:49
Shimadzu Biotech Axima Performance 2.9.3.20110624: Mode Linear, Power: 64, P.Ext. @ 2300 (bin 78)
%Int. 6.3 mV[sum= 1255 mV] Profiles 1-200 Smooth Av 50
874.6{r392}
1036.4{r371}
888.5{r351}
1050.3{r294}
1211.9{r322}
896.7{r189}
1374.0{r372}
m/z

(e)
